# Retinal imaging with *en face* and cross-sectional optical coherence tomography delineates outer retinal changes in cancer-associated retinopathy secondary to Merkel cell carcinoma

**DOI:** 10.1186/s12348-015-0053-0

**Published:** 2015-08-19

**Authors:** Nisreen K Mesiwala, Nathan Shemonski, Michelle G Sandrian, Ryan Shelton, Hiroshi Ishikawa, Hussein A Tawbi, Joel S Schuman, Stephen A Boppart, Leanne T Labriola

**Affiliations:** Department of Ophthalmology, UPMC Eye Center, Eye and Ear Institute, Ophthalmology and Visual Science Research Center, University of Pittsburgh School of Medicine, Pittsburgh, PA USA; Department of Electrical and Computer Engineering, Beckman Institute for Advanced Science and Technology, University of Illinois at Urbana-Champaign, Urbana, IL USA; University of Pittsburgh Cancer Institute, University of Pittsburgh Medical School, Pittsburgh, PA USA; Departments of Electrical and Computer Engineering, Bioengineering, and Medicine, University of Illinois at Urbana-Champaign, Urbana, IL USA; Department of Bioengineering, Swanson School of Engineering, University of Pittsburgh, Pittsburgh, PA USA; Department of Ophthalmology, Carle Foundation Hospital, Urbana, IL USA; Department of Surgery, University of Illinois Urbana-Champaign, Urbana, IL USA

**Keywords:** *En face*, Merkel, OCT, Paraneoplastic, Retinopathy

## Abstract

**Background:**

The study aims to correlate Fourier-domain optical coherence tomography (FD-OCT) with Goldmann visual field (GVF) to show the photoreceptor (PR) structure and function relationship in the first described case of cancer-associated retinopathy (CAR) from Merkel cell carcinoma.

**Findings:**

A case study of a patient with CAR who was imaged with serial GVF and FD-OCT over a 2-year period was carried out. *En face* images were created using a custom algorithm from the volumetric Fourier-domain OCT scans at the PR level. The areas of decreased PR reflectivity on the *en face* images were compared with GVF obtained at the same time point. Regions of reduced signal on *en face* scans corresponded with the position and shape of the GVF scotomas. Initially, the vision improved without PR changes. Cross-sectional OCTs showed early recovery of the outer nuclear layer and later improvement in the nerve fiber layer. Worsening vision corresponded with recurrence of the underlying cancer. Progressive global retinal atrophy was seen over time.

**Conclusions:**

Merkle cell carcinoma can cause CAR. Retinal function recovered without structural PR recovery. Transient vision improvements in treated CAR patients may be due to layers other than the PRs, but eventual vision decline results from significant progressive retinal atrophy.

## Findings

### Introduction

Advances in imaging have revolutionized the diagnosis and classification of many retinal diseases. Improved resolution with high-definition Fourier-domain optical coherence tomography (FD-OCT) can identify focal changes of the outer retinal layers. We compared cross-sectional and *en face* FD-OCT images of the outer retina to serial Goldmann visual fields (GVFs) to show the correspondence between the structure and function of the retina in a rare case of cancer-associated retinopathy (CAR).

### Methods

Institutional review board approval was obtained by The University of Pittsburgh Medical Center. Research adhered to the tenets of the declaration of Helsinki and was conducted in accordance with the Health Insurance Portability and Accountability Act. A 59-year-old female with CAR secondary to Merkel cell carcinoma was followed from March 2012 to February 2014. Over this time, GVF and FD-OCT scans (Spectralis HRA-OCT, Heidelberg, Germany, and Cirrus HD OCT, Zeiss, Dublin, CA) were obtained. The cross-sectional FD-OCT and retinal volume measurements from Spectralis with 73 raster lines consisting of 40,000 A-scans per second over a 5.8 × 4.35 mm area were compared. The *en face* images were created from the volumetric FD-OCT Cirrus data of 128 raster scans consisting of 512 A-scans per second over a 6 × 6 mm area using a custom automatic retinal pigment epithelium (RPE) alignment algorithm and semi-automated approach to produce the *en face* images with a thickness of 36 μm [1]. The same technician was used for all GVFs.

### Report of case

**Fig. 1:**
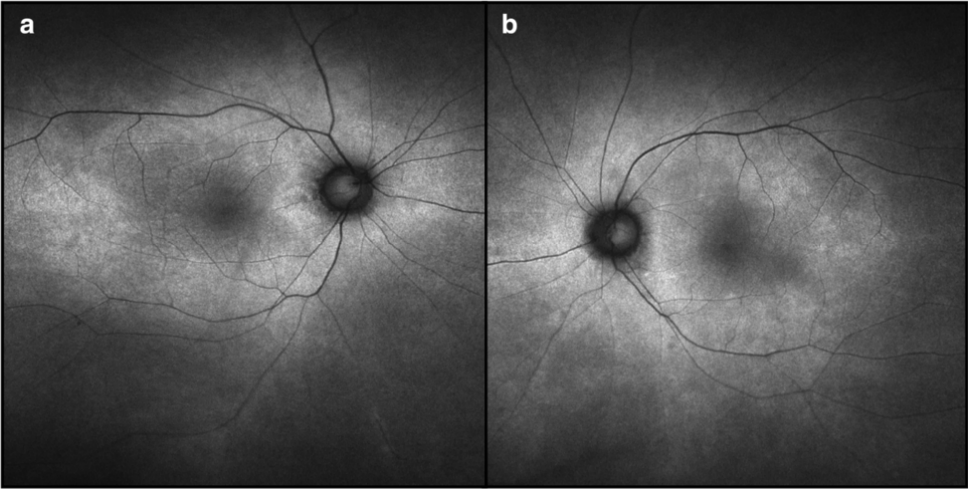
Blue-light autofluorescence from March 2012 of the right eye (**a**) and left eye (**b**) with abnormal hyperautofluorescence around arcades and in the peripapillary area and severe arteriole attenuation in both eyes, which supports the diagnosis of cancer-associated retinopathy

In March 2012, a 59-year-old African American female with no personal or family history of eye disease reported decreased vision, photoaversion, and photopsias for 1 month. Her medical history included diabetes type 2, hypercholesterolemia, hypertension, and gastroesophageal reflux disease, which were controlled with metformin, simvastatin, amlodipine, and hydrochlorothiazide and omeprazole, respectively. Best-corrected visual acuity (VA) was 20/30 in her right eye (OD) and 20/40 in her left eye (OS). She failed the Ishihara color testing. The exam was normal except for severe retinal arteriolar attenuation and mild peripheral pigmentation in both eyes (OU). FAF showed hyperautofluorescence around the arcades (Fig. 1). One month later, vision was 20/30 OD and count fingers at 3 ft OS. After a detailed discussion on the side effects of steroids, she was started on 1 mg/kg of oral prednisone for presumed paraneoplastic retinopathy. This diagnosis was supported by the electroretinogram findings of a non-detectable scotopic response in both eyes and a non-detectable photopic response OD with a severely diminished amplitude (a-wave – 12.34 μV and b-wave 25.55 μV) and delayed implicit time (a-wave 21 ms and b-wave 31 ms) OS. Within 2 months, vision improved to 20/30 OD and 20/70 OS.

Ophthalmic referral for systemic workup revealed a right thigh mass with high-grade Merkel cell carcinoma (MCC) and positive inguinal nodes, consistent with a stage III carcinoma. Blood work for anti-retinal antibody testing (Oregon Health Science University) was negative. With approval from her oncologist, she was kept on oral steroids. The patient received four cycles of carboplatin and etoposide with excellent response showing shrinkage of her nodal disease and underwent surgical resection of the tumor. She missed her next follow-up appointments due to a submassive pulmonary embolism requiring initial ICU admission but eventually was discharged in stable condition on warfarin. Her internist tapered her steroids.

In April 2013, while on 20 mg of prednisone, she returned with several months of decreased vision and VA 20/60 OD and 20/200 OS. A PET scan revealed cancer recurrence. She was admitted to the hospital for intravenous methylprednisolone 1 g/day for 3 days and IgG 1 mg/kg for 2 days then restarted chemotherapy (six cycles of carboplatin/etoposide). Vision improved to 20/60 OS. Steroids were tapered to 40 mg of prednisone, and chemotherapy was completed in August 2013. Vision then was 20/60 OD and 20/70 OS.

Three months later, she returned to the eye clinic with count fingers vision at 5 ft OD and 20/200 OS. A repeat PET scan showed a new area of cancer in her retroperitoneal fossa. She was given an injection of intravitreal triamcinolone OD and continued on oral steroids. In 1 month, vision improved to 20/200 OD and 20/80 OS, but intraocular pressure increased by 15 mmHg OD. The glaucoma service recommended combined phacoemulsification and Ahmed valve placement OD, which would have enabled her to continue local injections. However, at the end of February 2014, she showed further metastasis of her cancer to her kidney and lung, and 1 month later, she died of gram-negative rod urosepsis resulting in multi-organ failure. The patient was deceased and could not offer her consent to reporting the case.

### Results

**Fig. 2:**
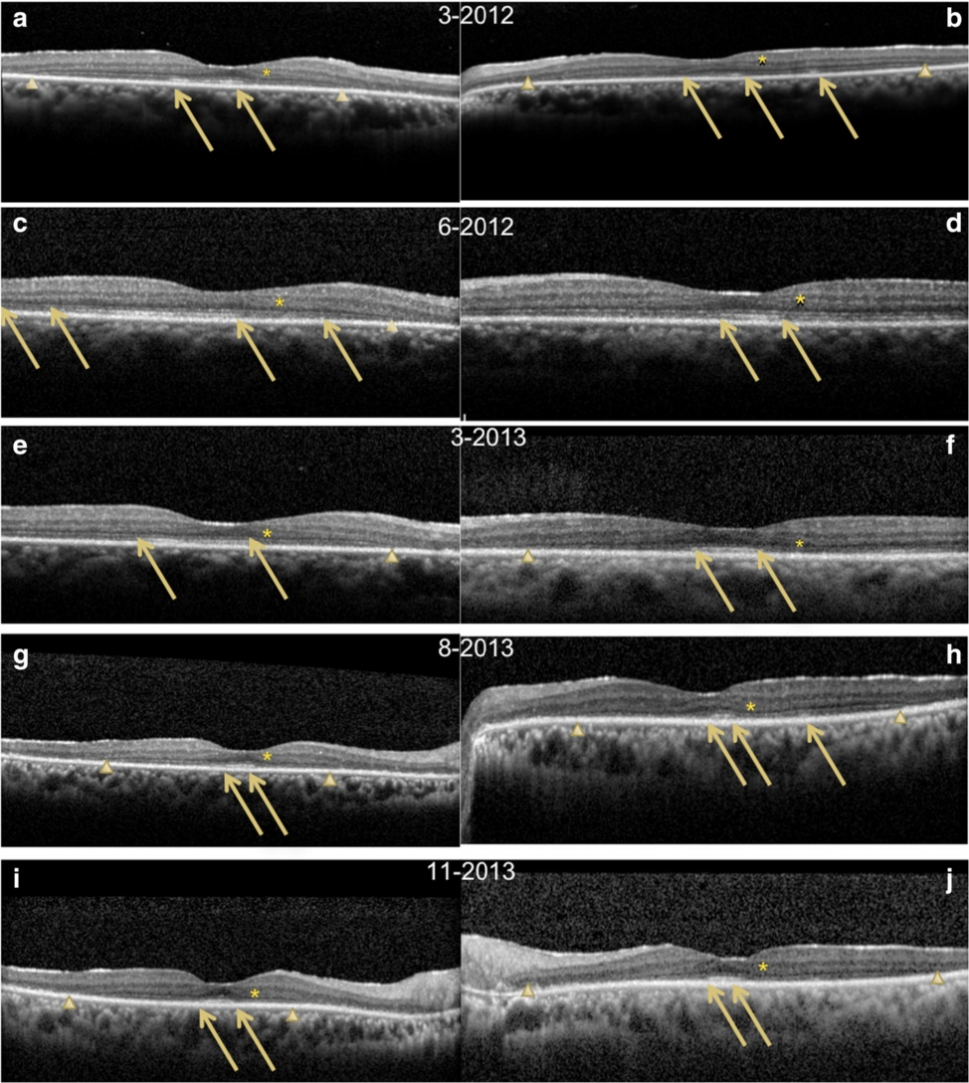
Fourier-domain cross-sectional optical coherence tomography (Spectralis) that shows initial disruption in the outer nuclear layer (ONL) and external limiting membrane (ELM) and decreases in reflectivity of the inner segment ellipsoid (ISe) in the **a** right eye and **b** left eye on March 2012. *Arrows* point to changes in the ISe, and *arrowheads* show tapering of the ONL. **c**, **d** All layers recover in the immediate follow-up visit on June 2012. **e**–**j** FD-OCT shows loss of the ONL and progressive decrease in reflectivity of the ISe with eventual loss of the layer in the final images. **i**, **j** Abnormal thickening of the nerve fiber layer. The ONL shows abnormal hyperreflectivity throughout the inner half on all images (*asterisk*)

**Fig. 3:**
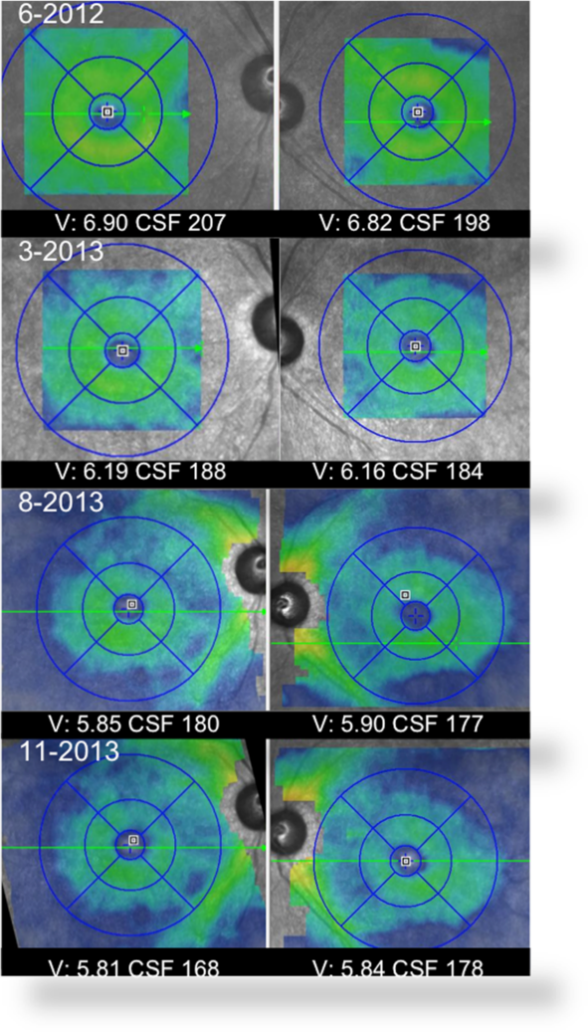
The volume map (Spectralis) from each visit shows progressive retinal atrophy. Right eye volume (*V*) on the listed dates is 6.90, 6.19, 5.85, and 5.81 mm^3^, and the left eye volume is 6.82, 6.16, 5.90, and 5.84 mm^3^. Central subfield thickness (CSF) is recorded on each image

**Fig. 4:**
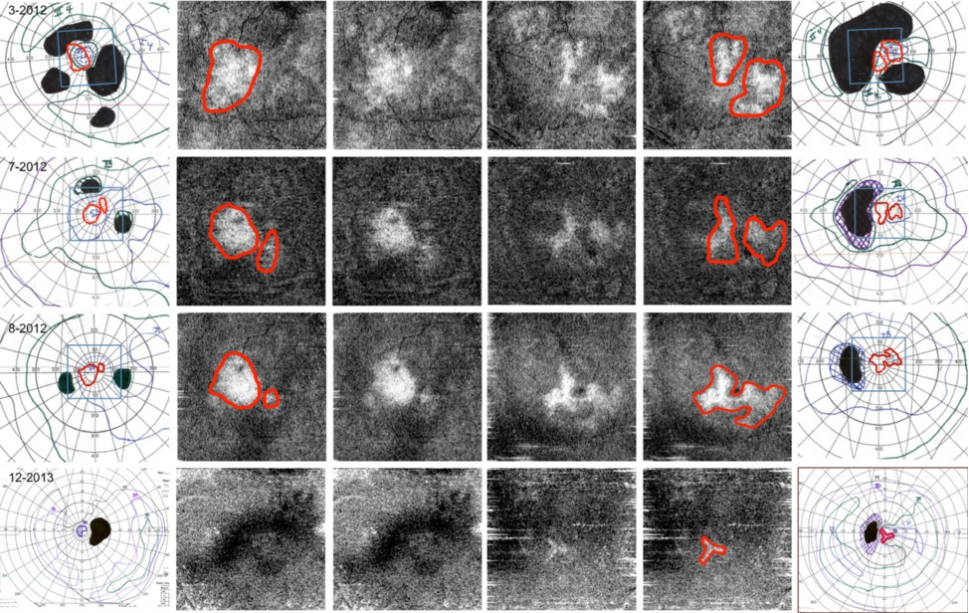
The *outer columns* show Goldmann visual fields (GVF) from the right eye and left eye. The *second* and *fifth columns* shows the *en face* OCT (Cirrus) scan with the intact central area of photoreceptors (PRs) delineated with a manually drawn *red line*. The *red line* from *en face* images has been transposed onto the GVF images on the corresponding dates. The *middle columns* show the original *en face* images. Initially, the size and shape of the PRs correspond to the preserved island of central vision, but as the GVF improves, the area of intact PRs declines. Eventually, there is complete loss from the PR signal in the right eye, which corresponds to a severely depressed GVF on December 2013 seen as restriction of the I4 isopter to the center 5°. The left eye also shows severe vision loss on the final GVF. The poor quality of the later *en face* images is due to increasing artifact when generating the *en face* images due to the retina thinning over time. (The *en face* images on July 2012 were created with a modified method using linear interpolation of the RPE slice since the automated technique was not available for these images)

The cross-sectional FD-OCT initially showed recovery of the outer retinal layers (Fig. 2). But overtime, thinning developed and the retina became atrophic as reflected in the retinal volume measurements in Fig. 3. The comparison of the GVF to the *en face* FD-OCT (Fig. 4) shows a striking correspondence between the locations of the visual field scotomas and the loss of photoreceptor (PR) reflectivity, with initial testing. Early treatment results in a transient reduction in the size and number of the GVF scotomas, but as the scotomas improve, there is no corresponding improvement in PR signal. Throughout the follow-up of this patient, subjective reports and objective evidence of worsening visual function correlated with recurrence of the underlying cancer and initiated a change to the systemic treatment. The end result was severe vision impairment with reduced visual acuity, decreased visual fields (with the I4e isopter limited to the center 10° of vision in the right eye and 40° × 5° cecocentral area of vision in the left eye), and overall retinal atrophy.

### Discussion

Cancer-associated retinopathy (CAR) is an aberrant immune response to a cancer, reported in lung (particularly small cell lung), prostate, breast, colon and uterine cancer, as well as, in lymphoma, that creates destructive autoantibodies that bind to and target healthy retina cells for destruction [2]. To our knowledge, this is the first case of CAR developing from MCC. MCC is a high-grade neuroendocrine tumor that biologically behaves in a similar fashion to small cell lung cancer, which might explain the occurrence of a paraneoplastic process [3]. MCC usually involves sun-exposed areas and has been reported with eyelid cancer [4]. There have been case reports of metastatic MCC to the iris, ciliary body, choroid, and superior rectus muscle [4–8]. MCC occurs at a higher incidence in the setting of long-standing immune suppression and is clearly correlated with a novel polyomavirus termed the Merkel cell virus [3]. In the opinion of the oncologist, the short course of oral steroids could not have contributed to the diagnosis of MCC and the oral steroid use in this case was approved and closely monitored; however, each case of CAR should be independently reviewed for the appropriateness of immunosuppression in the setting of a carcinoma.

MCC is aggressive with early metastasis and a widespread invasive disease in 27–31 % of patients [3]. The 3-year survival prognosis is 34 % [3]. Timely diagnosis and close monitoring for recurrence is critical. Our patient’s vision decline was the initial finding that initiated the workup for the diagnosis of her cancer, as well as with her later recurrences. Visual testing would be a useful marker in other patients who share this close interplay between antigen presentation by tumor and vision function.

In this case, the use of OCT showed a structural correlation to our patient’s functional visual decline. The initial improvement in visual acuity seemed to correspond with an improvement on the cross-sectional OCT images, in regard to an increase in the extent of the outer nuclear layer (ONL) and improvement of the reflection of the inner segment ellipsoid (ISe) junction (Fig. 2a–d). Previous studies in rhesus monkeys [9] and in vitro [10] show that PR outer segments are able to regenerate and reorganize. So this early subtle change in reflectivity in our patient may have indicated the beginning of a structural recovery in the PRs. The suggestion that PRs can recover is also supported by another case of CAR after treatment with rituximab [11]. It is important to note that reflectivity can be misleading [12], but we believe that this limitation did not affect our results since the findings were reproducible (Fig. 2). Furthermore, the changes on the *en face* OCT scan provided an even better analysis of the structure and functional relationship in our case. Previous reports of *en face* imaging have shown a positive correlation between *en face* outer retinal structure and visual acuity [13, 14].

Using this standard, the decrease in reflectivity on the *en face* scan should correlate with a decrease in visual acuity, so it is unclear why the visual function in our patient improved initially despite a constricting island of PR signal. One explanation could be that the *en face* OCT images would have eventually improved if our patient stayed in remission longer, creating a mismatch between the function and structural recovery. In this case, timely initiation of steroids may have halted or reduced the sensitivity of anti-retinal antibodies in attacking the retina to allow for vision recovery, but not in time to prevent changes to the reflectivity of the PRs on imaging. There have been other reports of vision function improving even without obvious signs of structural recovery [15, 16]. With this reasoning, the PRs can reorganize and restore function even though the reflectivity is abnormal. Of course, there are limitations to the interpretation of *en face* image reflections. The appearance of the PRs on OCT can change over minutes to hours because of the changes in the property of light as it passes through the PR outer segment and RPE interface; therefore, further studies should be done.

Another explanation may be that the visual recovery was due to recovery of visual function in layers other than the PRs. In our case, it is important to highlight the changes in the ONL and nerve fiber layer (NFL). The ONL seems to improve early in the course of the disease. The transient change layers other than the PRs may explain why the visual recovery was only temporary, and the redundancy in the connections between the axons of the inner nuclear layer and ONL to the PRs may explain the visual field improvements in this case.

Overall, the severe progressive atrophy of the retina documented on the OCT volume maps explains the overall poor vision outcome and may explain why early vision recovery cannot be sustained or recovered in cases of chronic CAR.

In conclusion, the initial correlation of the GVF scotomas and the *en face* PR structure in this patient is striking, and more research should be done to elucidate the accuracy and reproducibility of the relationship of structure and function in cases of progressive retinal diseases. This paper highlights the role of *en face* OCT in aiding diagnosis and guiding treatment in patients with CAR. In light of this case, MCC should be recognized as a serious and devastating cause of paraneoplastic retinopathy.

## Abbreviations

CAR: cancer-associated retinopathy
FD-OCT: Fourier-domain optical coherence tomography
GVF: Goldmann visual field
ISe: inner segment ellipsoid
MCC: Merkel cell carcinoma
NFL: nerve fiber layer
OD: right eye
ONL: outer nuclear layer
OS: left eye
OU: both eyes
PR: photoreceptor
VA: visual acuity
